# Crystal structure of {3-[3,5-bis­(2,6-di­methyl­phen­yl)-1,2-phenyl­ene]-1-(2,6,2′′,6′′-tetra­methyl-1,1′:3′,1′′-ter­phen­yl-5′-yl)imidazol-2-yl­idene}chlorido­(η^6^-*p*-cymene)ruthenium(II) benzene disolvate

**DOI:** 10.1107/S160053681402399X

**Published:** 2014-11-08

**Authors:** Shohei Sase, Yuriko Ikehara, Kei Goto

**Affiliations:** a Department of Chemistry, Graduate School of Science and Engineering, Tokyo Instiute of Technology, Ookayama, Meguro-ku, Tokyo, 152-8551, Japan

**Keywords:** crystal structure, ruthenium(II) complex, ruthenacycle, *N*-heterocyclic carbene

## Abstract

The title compound, [Ru(C_47_H_43_N_2_)Cl(C_10_H_14_)]·2C_6_H_6_, crystallized with two independent mol­ecules of benzene. One of the *N*-aryl moieties of the *N*-heterocyclic carbene (NHC) ligand underwent cyclo­metallation to form a five-membered ruthenacycle. The complex has a three-legged piano-stool structure with two C atoms incorporated in the five-membered ruthenacycle and a Cl atom as legs. The ruthenacycle is essentially coplanar with the imidazole ring of the NHC ligand, making a dihedral angle of 0.85 (8)°.

## Related literature   

For related cyclo­metalated ruthenium complexes bearing NHC and η^6^-arene ligands, see: Hong *et al.* (2007 ▶); Karabıyık *et al.* (2008 ▶); Zhang *et al.* (2009 ▶). For Pd complexes bearing the NHC ligands utilized in this study, see: Yamashita *et al.* (2005 ▶).
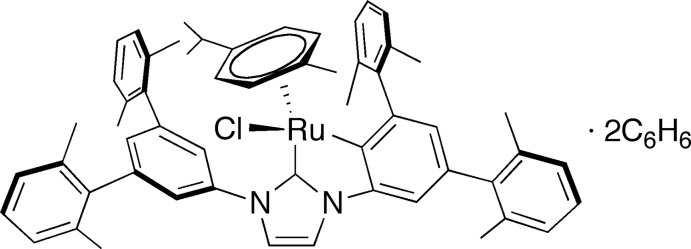


## Experimental   

### Crystal data  

[Ru(C_47_H_43_N_2_)Cl(C_10_H_14_)]·2C_6_H_6_
*M* *_r_* = 1062.78
Monoclinic, 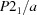

*a* = 12.9207 (12) Å
*b* = 19.2867 (17) Å
*c* = 22.977 (2) Å
β = 98.6792 (18)°
*V* = 5660.3 (9) Å^3^
*Z* = 4
Mo *K*α radiation
μ = 0.37 mm^−1^
*T* = 123 K
0.20 × 0.19 × 0.11 mm

### Data collection  

Rigaku Saturn CCD diffractometer
Absorption correction: multi-scan (*REQAB*; Jacobson, 1998 ▶) *T*_min_ = 0.930, *T*_max_ = 0.961
22748 measured reflections
12805 independent reflections
10644 reflections with *I* > 2σ(*I*)
*R*_int_ = 0.016

### Refinement  

*R*[*F* ^2^ > 2σ(*F* ^2^)] = 0.032
*wR*(*F* ^2^) = 0.092
*S* = 1.09
12805 reflections
669 parameters
H-atom parameters constrained
Δρ_max_ = 0.55 e Å^−3^
Δρ_min_ = −0.95 e Å^−3^

### 

Data collection: *CrystalClear* (Rigaku, 2009 ▶); cell refinement: *CrystalClear*; data reduction: *CrystalClear*; program(s) used to solve structure: *SHELXS97* (Sheldrick, 2008 ▶); program(s) used to refine structure: *SHELXL97* (Sheldrick, 2008 ▶); molecular graphics: *Yadokari-XG 2009* (Kabuto *et al.*, 2009 ▶); software used to prepare material for publication: *Yadokari-XG 2009*.

## Supplementary Material

Crystal structure: contains datablock(s) I, global. DOI: 10.1107/S160053681402399X/is5379sup1.cif

Structure factors: contains datablock(s) I. DOI: 10.1107/S160053681402399X/is5379Isup2.hkl

**Fig. 1.**
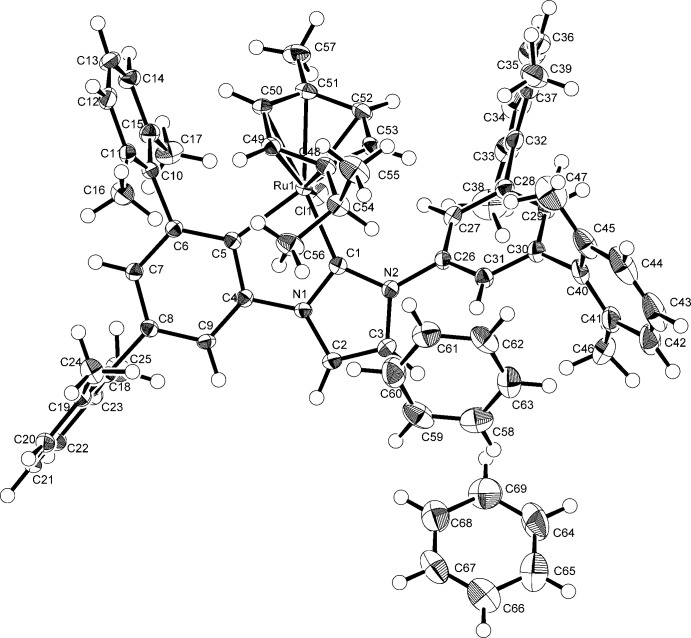
The asymmetric unit of the title compound with 50% probability displacement ellipsoids (arbitrary spheres for H atoms).

**Fig. 2.**
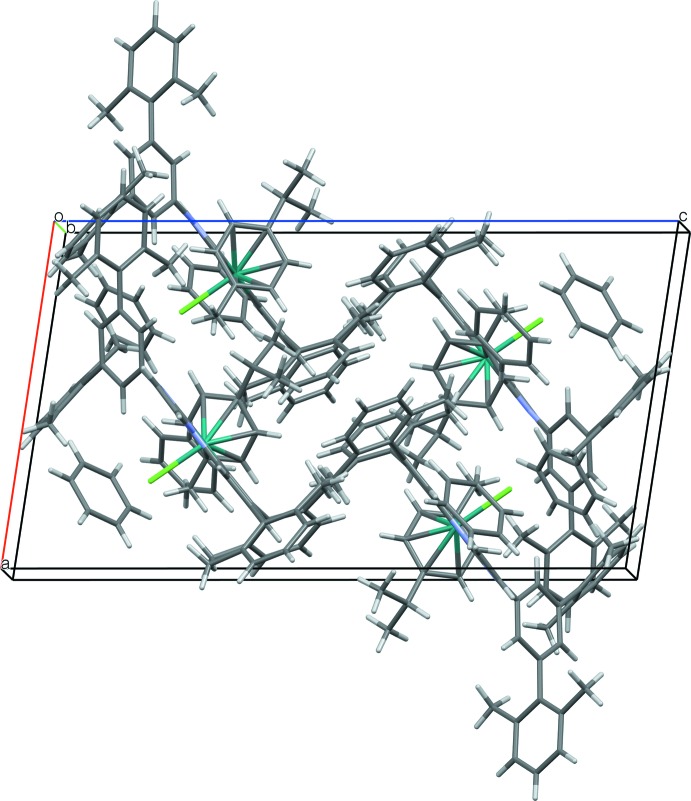
A packing diagram of the title compound viewed along the b axis.

. DOI: 10.1107/S160053681402399X/is5379fig1.tif
The asymmetric unit of the title compound with 50% probability displacement ellipsoids (arbitrary spheres for H atoms).

b . DOI: 10.1107/S160053681402399X/is5379fig2.tif
A packing diagram of the title compound viewed along the *b* axis.

CCDC reference: 1031852

Additional supporting information:  crystallographic information; 3D view; checkCIF report

## supplementary crystallographic information

### S1. Comment

Transition metal complexes bearing *N*-heterocyclic carbene (NHC) ligands have been broadly utilized in the catalytic reactions. It has been known that the catalytic performance largely depends on not only the electronic property of the ligand but also steric environment around the metal center. Since these properties can be tuned with changing the substituents on the nitrogen atoms and the carbene ring, a variety of NHCs have been synthesized. In the course of our studies on development of cavity-shaped ligands, we have reported an NHC based on a *m*-phenylene dendrimer framework and its palladium complexes (Yamashita *et al.*, 2005). Herein, the crystal structure of a ruthenium(II) complex bearing the cavity-shaped NHC is reported, where an aromatic ring on the nitrogen of the NHC ligand underwent cyclometallation to form a five-membered ruthenacycle. Related ruthenacycles bearing an NHC ligand have been reported in the literatures (Hong *et al.*, 2007; Karabıyık *et al.*, 2008; Zhang *et al.*, 2009).

The title compound was synthesized by the reaction of dichloro-(*p*-cymene)-ruthenium(II) dimer with 1,3-bis(2,2",6,6"-tetramethyl-*m*-terphenyl-5'-yl)imidazol-2-ylidene. The molecular structure of the title compound is shown in Fig. 1. It was found that one of *N*-aryl moieties underwent cyclometallation to form a ruthenacycle, which is almost coplanar with the imidazole ring. The bond length of Ru—C(carbene) is 2.0163 (15) Å, which is slightly shorter than that of Ru—C(arene) [2.1192 (14) Å].

### S2. Experimental

In a dry box under an argon atmosphere, dichloro-(*p*-cymeme)-ruthenium(II) (36.3 mg, 0.0593 mmol) was added to a solution of 1,3-bis(2,2",6,6"-tetramethyl-*m*-terphenyl-5'-yl)imidazol-2-ylidene (75.5 mg, 0.119 mmol) in benzene (1 ml) at room temperature. After the reaction mixture was stirred for 1 h at room temperature, the solution was evaporated *in vacuo* to afford orange solids, which was put out from the dry box. To the solids was added benzene, and the resulting mixture was subjected to centrifugal separation, and the supernatant was evaporated *in vacuo*. The resulting orange solids were separated by silica gel chromatography (hexane:ethyl acetate = 3:1) to afford the title compound (66.2 mg, 0.0731 mmol, 61%). Single crystals suitable for X-ray diffraction were obtained by recrystallization from a benzene/hexane solution.

### S3. Refinement

H atoms were treated as riding with C—H = 0.95–1.00 Å and with *U*_iso_(H) = 1.2 (1.5 for methyl groups) times *U*_eq_(C).

### Crystal data

**Table:**

[Ru(C_47_H_43_N_2_)Cl(C_10_H_14_)]·2C_6_H_6_	*Z* = 4
*M**_r_* = 1062.78	*F*(000) = 2232
Monoclinic, *P*2_1_/*a*	*D*_x_ = 1.247 Mg m^−^^3^
Hall symbol: -P 2yab	Mo *K*α radiation, λ = 0.71075 Å
*a* = 12.9207 (12) Å	θ = 3.1–27.5°
*b* = 19.2867 (17) Å	µ = 0.37 mm^−^^1^
*c* = 22.977 (2) Å	*T* = 123 K
β = 98.6792 (18)°	Block, yellow
*V* = 5660.3 (9) Å^3^	0.20 × 0.19 × 0.11 mm

### Data collection

**Table:**

Rigaku Saturn CCD diffractometer	12805 independent reflections
Radiation source: fine-focus sealed tube	10644 reflections with *I* > 2σ(*I*)
Graphite monochromator	*R*_int_ = 0.016
Detector resolution: 28.5714 pixels mm^-1^	θ_max_ = 27.5°, θ_min_ = 3.1°
ω scans	*h* = −16→14
Absorption correction: multi-scan (*REQAB*; Jacobson, 1998)	*k* = −25→23
*T*_min_ = 0.930, *T*_max_ = 0.961	*l* = −19→29
22748 measured reflections	

### Refinement

**Table:**

Refinement on *F*^2^	Primary atom site location: structure-invariant direct methods
Least-squares matrix: full	Secondary atom site location: difference Fourier map
*R*[*F*^2^ > 2σ(*F*^2^)] = 0.032	Hydrogen site location: inferred from neighbouring sites
*wR*(*F*^2^) = 0.092	H-atom parameters constrained
*S* = 1.09	*w* = 1/[σ^2^(*F*_o_^2^) + (0.0601*P*)^2^ + 0.2421*P*] where *P* = (*F*_o_^2^ + 2*F*_c_^2^)/3
12805 reflections	(Δ/σ)_max_ = 0.002
669 parameters	Δρ_max_ = 0.55 e Å^−^^3^
0 restraints	Δρ_min_ = −0.95 e Å^−^^3^

### Special details

**Table:**

Geometry. All e.s.d.'s (except the e.s.d. in the dihedral angle between two l.s. planes) are estimated using the full covariance matrix. The cell e.s.d.'s are taken into account individually in the estimation of e.s.d.'s in distances, angles and torsion angles; correlations between e.s.d.'s in cell parameters are only used when they are defined by crystal symmetry. An approximate (isotropic) treatment of cell e.s.d.'s is used for estimating e.s.d.'s involving l.s. planes.
Refinement. Refinement of *F*^2^ against ALL reflections. The weighted *R*-factor *wR* and goodness of fit *S* are based on *F*^2^, conventional *R*-factors *R* are based on *F*, with *F* set to zero for negative *F*^2^. The threshold expression of *F*^2^ > σ(*F*^2^) is used only for calculating *R*-factors(gt) *etc*. and is not relevant to the choice of reflections for refinement. *R*-factors based on *F*^2^ are statistically about twice as large as those based on *F*, and *R*- factors based on ALL data will be even larger.

### Fractional atomic coordinates and isotropic or equivalent isotropic displacement parameters (Å^2^)

**Table:**

	*x*	*y*	*z*	*U*_iso_*/*U*_eq_	
Ru1	0.360440 (8)	−0.062895 (6)	0.706449 (5)	0.01560 (5)	
Cl1	0.25369 (3)	−0.05112 (2)	0.783182 (17)	0.02670 (9)	
C1	0.43058 (10)	0.02365 (8)	0.74243 (6)	0.0173 (3)	
N1	0.39048 (9)	0.08574 (7)	0.72351 (6)	0.0179 (2)	
C2	0.44408 (11)	0.14053 (8)	0.75316 (7)	0.0232 (3)	
H2	0.4297	0.1886	0.7475	0.028*	
C3	0.52060 (11)	0.11182 (9)	0.79160 (7)	0.0253 (3)	
H3	0.5716	0.1360	0.8182	0.030*	
N2	0.51176 (9)	0.04041 (7)	0.78549 (6)	0.0200 (3)	
C4	0.30243 (10)	0.08444 (8)	0.67846 (6)	0.0173 (3)	
C5	0.27103 (10)	0.01743 (8)	0.66026 (6)	0.0166 (3)	
C6	0.18454 (10)	0.01623 (8)	0.61421 (6)	0.0180 (3)	
C7	0.13737 (11)	0.07769 (8)	0.59093 (7)	0.0196 (3)	
H7	0.0794	0.0747	0.5602	0.023*	
C8	0.17223 (10)	0.14278 (8)	0.61109 (6)	0.0179 (3)	
C9	0.25706 (10)	0.14582 (8)	0.65617 (6)	0.0179 (3)	
H9	0.2834	0.1892	0.6714	0.021*	
C10	0.14148 (11)	−0.04913 (8)	0.58473 (7)	0.0197 (3)	
C11	0.17037 (12)	−0.06778 (8)	0.53053 (7)	0.0228 (3)	
C12	0.13424 (12)	−0.13031 (9)	0.50442 (7)	0.0283 (4)	
H12	0.1544	−0.1437	0.4679	0.034*	
C13	0.06951 (12)	−0.17299 (9)	0.53110 (8)	0.0317 (4)	
H13	0.0478	−0.2164	0.5139	0.038*	
C14	0.03644 (12)	−0.15230 (9)	0.58289 (8)	0.0302 (4)	
H14	−0.0108	−0.1809	0.6000	0.036*	
C15	0.07086 (11)	−0.09050 (9)	0.61051 (7)	0.0244 (3)	
C16	0.23756 (13)	−0.02156 (10)	0.49880 (8)	0.0310 (4)	
H16	0.2731	−0.0496	0.4722	0.046*	
H16A	0.2898	0.0017	0.5276	0.046*	
H16B	0.1935	0.0132	0.4759	0.046*	
C17	0.03219 (14)	−0.06806 (10)	0.66587 (9)	0.0345 (4)	
H17	−0.0212	−0.1007	0.6751	0.052*	
H17A	0.0017	−0.0216	0.6603	0.052*	
H17B	0.0908	−0.0672	0.6984	0.052*	
C18	0.12377 (10)	0.20829 (8)	0.58586 (6)	0.0179 (3)	
C19	0.15761 (10)	0.23791 (8)	0.53645 (7)	0.0214 (3)	
C20	0.11532 (11)	0.30149 (9)	0.51559 (7)	0.0252 (3)	
H20	0.1388	0.3225	0.4826	0.030*	
C21	0.03990 (12)	0.33414 (8)	0.54231 (7)	0.0267 (3)	
H21	0.0120	0.3774	0.5278	0.032*	
C22	0.00492 (12)	0.30360 (8)	0.59040 (7)	0.0249 (3)	
H22	−0.0476	0.3260	0.6084	0.030*	
C23	0.04557 (11)	0.24070 (8)	0.61270 (7)	0.0207 (3)	
C24	0.23403 (12)	0.20088 (10)	0.50395 (8)	0.0302 (4)	
H24	0.2988	0.1917	0.5310	0.045*	
H24A	0.2495	0.2299	0.4713	0.045*	
H24B	0.2035	0.1569	0.4884	0.045*	
C25	0.00462 (13)	0.20640 (10)	0.66344 (8)	0.0309 (4)	
H25	0.0553	0.2123	0.6994	0.046*	
H25A	−0.0060	0.1568	0.6551	0.046*	
H25B	−0.0621	0.2277	0.6689	0.046*	
C26	0.57476 (11)	−0.00688 (8)	0.82359 (6)	0.0196 (3)	
C27	0.52753 (11)	−0.05939 (8)	0.85101 (7)	0.0208 (3)	
H27	0.4537	−0.0650	0.8436	0.025*	
C28	0.58861 (11)	−0.10408 (8)	0.88957 (6)	0.0220 (3)	
C29	0.69716 (11)	−0.09314 (9)	0.90096 (7)	0.0236 (3)	
H29	0.7394	−0.1225	0.9281	0.028*	
C30	0.74393 (11)	−0.04028 (9)	0.87333 (7)	0.0226 (3)	
C31	0.68254 (11)	0.00313 (8)	0.83397 (6)	0.0217 (3)	
H31	0.7138	0.0392	0.8144	0.026*	
C32	0.53705 (12)	−0.16162 (9)	0.91814 (7)	0.0261 (3)	
C33	0.45692 (14)	−0.14639 (11)	0.95144 (8)	0.0367 (4)	
C34	0.40964 (16)	−0.20071 (13)	0.97768 (9)	0.0483 (5)	
H34	0.3556	−0.1908	1.0002	0.058*	
C35	0.43940 (17)	−0.26760 (13)	0.97159 (9)	0.0528 (6)	
H35	0.4070	−0.3038	0.9904	0.063*	
C36	0.51653 (16)	−0.28311 (11)	0.93820 (9)	0.0437 (5)	
H36	0.5362	−0.3301	0.9337	0.052*	
C37	0.56629 (13)	−0.23046 (10)	0.91084 (8)	0.0328 (4)	
C38	0.41956 (18)	−0.07368 (12)	0.96011 (11)	0.0526 (6)	
H38	0.3609	−0.0627	0.9292	0.079*	
H38A	0.4769	−0.0410	0.9581	0.079*	
H38B	0.3965	−0.0701	0.9987	0.079*	
C39	0.64693 (16)	−0.25022 (11)	0.87263 (9)	0.0447 (5)	
H39	0.6382	−0.2210	0.8373	0.067*	
H39A	0.6376	−0.2990	0.8611	0.067*	
H39B	0.7173	−0.2435	0.8947	0.067*	
C40	0.86022 (11)	−0.02824 (10)	0.88395 (7)	0.0286 (4)	
C41	0.90130 (13)	0.02343 (11)	0.92252 (9)	0.0379 (4)	
C42	1.00997 (15)	0.03608 (14)	0.92929 (11)	0.0569 (7)	
H42	1.0406	0.0702	0.9564	0.068*	
C43	1.07192 (14)	−0.00134 (15)	0.89639 (12)	0.0632 (8)	
H43	1.1449	0.0078	0.9009	0.076*	
C44	1.03048 (15)	−0.05101 (14)	0.85766 (12)	0.0568 (7)	
H44	1.0745	−0.0753	0.8351	0.068*	
C45	0.92334 (14)	−0.06656 (10)	0.85087 (10)	0.0392 (5)	
C46	0.83268 (18)	0.06602 (12)	0.95604 (11)	0.0526 (6)	
H46	0.7856	0.0947	0.9285	0.079*	
H46A	0.8764	0.0960	0.9841	0.079*	
H46B	0.7913	0.0352	0.9774	0.079*	
C47	0.87912 (17)	−0.12114 (13)	0.80810 (10)	0.0544 (6)	
H47	0.8523	−0.1596	0.8294	0.082*	
H47A	0.9341	−0.1383	0.7867	0.082*	
H47B	0.8220	−0.1014	0.7801	0.082*	
C48	0.49177 (11)	−0.09900 (8)	0.66471 (7)	0.0208 (3)	
C49	0.39614 (11)	−0.11007 (8)	0.62670 (6)	0.0205 (3)	
H49	0.3849	−0.0884	0.5891	0.025*	
C50	0.31645 (11)	−0.15338 (8)	0.64406 (7)	0.0214 (3)	
H50	0.2562	−0.1643	0.6165	0.026*	
C51	0.32649 (12)	−0.17976 (8)	0.70106 (7)	0.0244 (3)	
C52	0.42221 (12)	−0.16792 (8)	0.74033 (7)	0.0242 (3)	
H52	0.4303	−0.1857	0.7793	0.029*	
C53	0.50315 (11)	−0.13062 (8)	0.72169 (7)	0.0228 (3)	
H53	0.5676	−0.1260	0.7474	0.027*	
C54	0.58173 (12)	−0.05819 (9)	0.64685 (8)	0.0265 (4)	
H54	0.6208	−0.0365	0.6832	0.032*	
C55	0.65677 (14)	−0.10768 (11)	0.62205 (10)	0.0421 (5)	
H55	0.6816	−0.1429	0.6516	0.063*	
H55A	0.7167	−0.0815	0.6120	0.063*	
H55B	0.6202	−0.1303	0.5866	0.063*	
C56	0.54651 (13)	−0.00056 (10)	0.60360 (9)	0.0349 (4)	
H56	0.5142	−0.0207	0.5660	0.052*	
H56A	0.6071	0.0275	0.5974	0.052*	
H56B	0.4954	0.0288	0.6194	0.052*	
C57	0.23787 (14)	−0.21838 (10)	0.72272 (9)	0.0363 (4)	
H57	0.1762	−0.2178	0.6921	0.055*	
H57A	0.2209	−0.1959	0.7583	0.055*	
H57B	0.2591	−0.2664	0.7317	0.055*	
C58	0.83002 (16)	0.17078 (11)	0.78449 (10)	0.0447 (5)	
H58	0.8362	0.2039	0.8154	0.054*	
C59	0.75182 (15)	0.17741 (11)	0.73663 (11)	0.0462 (5)	
H59	0.7041	0.2151	0.7347	0.055*	
C60	0.74318 (14)	0.12989 (12)	0.69222 (10)	0.0482 (5)	
H60	0.6895	0.1347	0.6593	0.058*	
C61	0.81209 (16)	0.07480 (11)	0.69479 (10)	0.0441 (5)	
H61	0.8056	0.0416	0.6639	0.053*	
C62	0.89048 (15)	0.06814 (10)	0.74252 (10)	0.0398 (5)	
H62	0.9384	0.0306	0.7444	0.048*	
C63	0.89893 (14)	0.11590 (12)	0.78721 (9)	0.0410 (5)	
H63	0.9525	0.1111	0.8202	0.049*	
C64	0.7807 (2)	0.28850 (15)	0.92676 (11)	0.0667 (7)	
H64	0.8077	0.2625	0.9608	0.080*	
C65	0.83034 (18)	0.34796 (16)	0.91335 (11)	0.0654 (7)	
H65	0.8914	0.3631	0.9384	0.079*	
C66	0.79277 (18)	0.38599 (14)	0.86393 (11)	0.0571 (6)	
H66	0.8276	0.4270	0.8547	0.068*	
C67	0.70389 (18)	0.36358 (13)	0.82818 (10)	0.0513 (6)	
H67	0.6771	0.3892	0.7939	0.062*	
C68	0.65344 (18)	0.30398 (13)	0.84197 (10)	0.0524 (6)	
H68	0.5918	0.2891	0.8172	0.063*	
C69	0.6915 (2)	0.26635 (14)	0.89088 (10)	0.0594 (6)	
H69	0.6568	0.2253	0.9001	0.071*	

### Atomic displacement parameters (Å^2^)

**Table:**

	*U*^11^	*U*^22^	*U*^33^	*U*^12^	*U*^13^	*U*^23^
Ru1	0.01645 (7)	0.01408 (7)	0.01647 (7)	0.00035 (4)	0.00309 (4)	0.00014 (4)
Cl1	0.02472 (18)	0.0330 (2)	0.02461 (19)	−0.00300 (15)	0.01080 (14)	−0.00258 (16)
C1	0.0160 (6)	0.0181 (7)	0.0184 (7)	−0.0004 (5)	0.0047 (5)	0.0005 (6)
N1	0.0176 (5)	0.0143 (6)	0.0214 (6)	−0.0012 (5)	0.0016 (5)	0.0002 (5)
C2	0.0218 (7)	0.0170 (8)	0.0295 (8)	−0.0033 (6)	−0.0006 (6)	−0.0009 (6)
C3	0.0224 (7)	0.0217 (8)	0.0294 (8)	−0.0051 (6)	−0.0033 (6)	0.0001 (7)
N2	0.0166 (5)	0.0195 (7)	0.0229 (6)	−0.0014 (5)	0.0000 (5)	0.0011 (5)
C4	0.0150 (6)	0.0187 (7)	0.0184 (7)	0.0000 (5)	0.0028 (5)	−0.0005 (6)
C5	0.0144 (6)	0.0171 (7)	0.0188 (7)	0.0016 (5)	0.0040 (5)	0.0009 (6)
C6	0.0164 (6)	0.0175 (7)	0.0199 (7)	0.0009 (5)	0.0027 (5)	−0.0021 (6)
C7	0.0168 (6)	0.0189 (8)	0.0223 (7)	0.0001 (5)	0.0007 (5)	−0.0013 (6)
C8	0.0167 (6)	0.0172 (7)	0.0202 (7)	0.0013 (5)	0.0041 (5)	0.0008 (6)
C9	0.0173 (6)	0.0140 (7)	0.0224 (7)	−0.0025 (5)	0.0033 (5)	−0.0019 (6)
C10	0.0171 (6)	0.0162 (7)	0.0243 (8)	0.0016 (5)	−0.0017 (5)	−0.0011 (6)
C11	0.0218 (7)	0.0220 (8)	0.0226 (8)	0.0037 (6)	−0.0033 (6)	−0.0023 (6)
C12	0.0264 (7)	0.0283 (9)	0.0272 (8)	0.0065 (7)	−0.0058 (6)	−0.0081 (7)
C13	0.0238 (7)	0.0217 (9)	0.0452 (11)	0.0008 (6)	−0.0089 (7)	−0.0106 (8)
C14	0.0190 (7)	0.0236 (9)	0.0462 (10)	−0.0043 (6)	−0.0011 (6)	−0.0028 (8)
C15	0.0171 (7)	0.0227 (8)	0.0330 (9)	−0.0003 (6)	0.0021 (6)	−0.0020 (7)
C16	0.0381 (9)	0.0307 (10)	0.0248 (8)	0.0040 (7)	0.0070 (7)	−0.0002 (7)
C17	0.0273 (8)	0.0347 (11)	0.0444 (11)	−0.0065 (7)	0.0144 (8)	−0.0053 (8)
C18	0.0153 (6)	0.0151 (7)	0.0221 (7)	−0.0017 (5)	−0.0007 (5)	−0.0006 (6)
C19	0.0165 (6)	0.0214 (8)	0.0255 (8)	−0.0034 (6)	0.0006 (5)	0.0012 (6)
C20	0.0246 (7)	0.0225 (8)	0.0270 (8)	−0.0056 (6)	−0.0012 (6)	0.0070 (7)
C21	0.0290 (8)	0.0147 (8)	0.0325 (9)	0.0006 (6)	−0.0079 (6)	0.0012 (6)
C22	0.0227 (7)	0.0206 (8)	0.0300 (8)	0.0043 (6)	−0.0004 (6)	−0.0043 (7)
C23	0.0198 (6)	0.0185 (8)	0.0231 (7)	−0.0008 (6)	0.0009 (5)	−0.0024 (6)
C24	0.0257 (7)	0.0361 (10)	0.0309 (9)	0.0011 (7)	0.0109 (6)	0.0071 (8)
C25	0.0318 (8)	0.0326 (10)	0.0308 (9)	0.0061 (7)	0.0129 (7)	0.0020 (8)
C26	0.0191 (6)	0.0205 (8)	0.0182 (7)	0.0020 (6)	−0.0001 (5)	−0.0002 (6)
C27	0.0183 (7)	0.0249 (9)	0.0189 (7)	−0.0021 (6)	0.0018 (5)	−0.0021 (6)
C28	0.0251 (7)	0.0229 (8)	0.0179 (7)	−0.0030 (6)	0.0028 (5)	−0.0005 (6)
C29	0.0241 (7)	0.0271 (9)	0.0186 (7)	0.0008 (6)	0.0002 (5)	0.0036 (6)
C30	0.0186 (7)	0.0267 (8)	0.0221 (7)	0.0014 (6)	0.0019 (5)	0.0023 (6)
C31	0.0202 (7)	0.0237 (8)	0.0211 (7)	−0.0001 (6)	0.0027 (5)	0.0028 (6)
C32	0.0282 (7)	0.0287 (9)	0.0194 (7)	−0.0089 (7)	−0.0025 (6)	0.0039 (6)
C33	0.0362 (9)	0.0454 (12)	0.0292 (9)	−0.0133 (8)	0.0074 (7)	0.0038 (8)
C34	0.0471 (11)	0.0587 (15)	0.0410 (11)	−0.0199 (10)	0.0129 (9)	0.0089 (10)
C35	0.0586 (13)	0.0576 (15)	0.0404 (11)	−0.0301 (12)	0.0013 (9)	0.0178 (11)
C36	0.0582 (12)	0.0301 (11)	0.0373 (10)	−0.0138 (9)	−0.0108 (9)	0.0090 (9)
C37	0.0388 (9)	0.0298 (10)	0.0260 (8)	−0.0062 (7)	−0.0075 (7)	0.0050 (7)
C38	0.0590 (14)	0.0564 (15)	0.0507 (14)	−0.0052 (11)	0.0347 (11)	−0.0036 (11)
C39	0.0556 (12)	0.0301 (11)	0.0474 (12)	0.0063 (9)	0.0046 (9)	0.0033 (9)
C40	0.0173 (7)	0.0355 (10)	0.0318 (9)	0.0001 (7)	0.0005 (6)	0.0162 (8)
C41	0.0262 (8)	0.0402 (11)	0.0429 (11)	−0.0048 (8)	−0.0090 (7)	0.0143 (9)
C42	0.0320 (10)	0.0599 (15)	0.0706 (16)	−0.0177 (10)	−0.0189 (10)	0.0320 (13)
C43	0.0163 (8)	0.0821 (19)	0.0888 (19)	−0.0036 (10)	0.0005 (10)	0.0576 (16)
C44	0.0256 (9)	0.0742 (17)	0.0743 (17)	0.0132 (10)	0.0195 (10)	0.0453 (14)
C45	0.0251 (8)	0.0465 (12)	0.0486 (12)	0.0107 (8)	0.0138 (8)	0.0244 (9)
C46	0.0505 (12)	0.0491 (14)	0.0500 (14)	−0.0040 (10)	−0.0188 (10)	−0.0097 (10)
C47	0.0557 (12)	0.0629 (16)	0.0493 (13)	0.0212 (12)	0.0230 (10)	0.0023 (11)
C48	0.0207 (7)	0.0190 (8)	0.0236 (8)	0.0042 (6)	0.0065 (5)	−0.0006 (6)
C49	0.0268 (7)	0.0180 (7)	0.0172 (7)	0.0051 (6)	0.0048 (5)	−0.0016 (6)
C50	0.0251 (7)	0.0147 (7)	0.0240 (8)	0.0017 (6)	0.0022 (6)	−0.0043 (6)
C51	0.0301 (8)	0.0145 (8)	0.0288 (8)	0.0008 (6)	0.0052 (6)	0.0009 (6)
C52	0.0318 (8)	0.0169 (8)	0.0237 (8)	0.0036 (6)	0.0037 (6)	0.0034 (6)
C53	0.0236 (7)	0.0207 (8)	0.0237 (7)	0.0085 (6)	0.0020 (6)	0.0003 (6)
C54	0.0236 (7)	0.0279 (9)	0.0299 (9)	0.0016 (6)	0.0097 (6)	0.0023 (7)
C55	0.0354 (9)	0.0408 (12)	0.0554 (12)	0.0127 (8)	0.0243 (8)	0.0096 (10)
C56	0.0330 (8)	0.0286 (10)	0.0464 (11)	−0.0003 (7)	0.0171 (7)	0.0088 (8)
C57	0.0435 (10)	0.0251 (10)	0.0418 (11)	−0.0091 (8)	0.0104 (8)	0.0042 (8)
C58	0.0510 (11)	0.0411 (12)	0.0474 (12)	−0.0113 (10)	0.0248 (9)	−0.0020 (10)
C59	0.0352 (10)	0.0376 (12)	0.0700 (15)	0.0092 (9)	0.0219 (10)	0.0166 (11)
C60	0.0313 (9)	0.0533 (14)	0.0570 (13)	−0.0019 (9)	−0.0033 (9)	0.0121 (11)
C61	0.0462 (11)	0.0411 (12)	0.0456 (12)	−0.0048 (9)	0.0087 (9)	−0.0017 (9)
C62	0.0360 (10)	0.0383 (12)	0.0486 (13)	0.0083 (8)	0.0180 (9)	0.0152 (9)
C63	0.0307 (9)	0.0577 (14)	0.0353 (10)	−0.0052 (9)	0.0072 (7)	0.0154 (9)
C64	0.0867 (18)	0.076 (2)	0.0354 (12)	0.0155 (15)	0.0030 (12)	0.0201 (13)
C65	0.0512 (13)	0.089 (2)	0.0540 (15)	0.0042 (13)	0.0020 (11)	0.0047 (14)
C66	0.0536 (12)	0.0629 (17)	0.0596 (15)	0.0141 (12)	0.0246 (11)	0.0122 (12)
C67	0.0609 (13)	0.0560 (15)	0.0392 (11)	0.0254 (11)	0.0150 (10)	0.0106 (10)
C68	0.0587 (13)	0.0576 (15)	0.0404 (12)	0.0143 (11)	0.0057 (10)	−0.0068 (11)
C69	0.0860 (17)	0.0583 (16)	0.0365 (12)	−0.0063 (13)	0.0177 (11)	0.0002 (11)

### Geometric parameters (Å, º)

**Table:**

Ru1—C1	2.0163 (15)	C34—H34	0.9500
Ru1—C5	2.1192 (14)	C35—C36	1.379 (3)
Ru1—C49	2.1572 (14)	C35—H35	0.9500
Ru1—C48	2.1852 (14)	C36—C37	1.401 (2)
Ru1—C53	2.2437 (14)	C36—H36	0.9500
Ru1—C52	2.2713 (16)	C37—C39	1.509 (3)
Ru1—C50	2.2754 (15)	C38—H38	0.9800
Ru1—C51	2.2961 (16)	C38—H38A	0.9800
Ru1—Cl1	2.4076 (4)	C38—H38B	0.9800
C1—N1	1.350 (2)	C39—H39	0.9800
C1—N2	1.3675 (18)	C39—H39A	0.9800
N1—C2	1.385 (2)	C39—H39B	0.9800
N1—C4	1.4180 (17)	C40—C41	1.385 (3)
C2—C3	1.342 (2)	C40—C45	1.406 (3)
C2—H2	0.9500	C41—C42	1.411 (2)
C3—N2	1.387 (2)	C41—C46	1.503 (3)
C3—H3	0.9500	C42—C43	1.384 (4)
N2—C26	1.4303 (19)	C42—H42	0.9500
C4—C9	1.384 (2)	C43—C44	1.361 (4)
C4—C5	1.399 (2)	C43—H43	0.9500
C5—C6	1.4186 (18)	C44—C45	1.402 (3)
C6—C7	1.401 (2)	C44—H44	0.9500
C6—C10	1.498 (2)	C45—C47	1.494 (3)
C7—C8	1.389 (2)	C46—H46	0.9800
C7—H7	0.9500	C46—H46A	0.9800
C8—C9	1.3912 (19)	C46—H46B	0.9800
C8—C18	1.488 (2)	C47—H47	0.9800
C9—H9	0.9500	C47—H47A	0.9800
C10—C11	1.400 (2)	C47—H47B	0.9800
C10—C15	1.408 (2)	C48—C49	1.418 (2)
C11—C12	1.396 (2)	C48—C53	1.432 (2)
C11—C16	1.507 (2)	C48—C54	1.511 (2)
C12—C13	1.381 (2)	C49—C50	1.429 (2)
C12—H12	0.9500	C49—H49	0.9500
C13—C14	1.382 (3)	C50—C51	1.393 (2)
C13—H13	0.9500	C50—H50	0.9500
C14—C15	1.391 (2)	C51—C52	1.435 (2)
C14—H14	0.9500	C51—C57	1.512 (2)
C15—C17	1.499 (2)	C52—C53	1.389 (2)
C16—H16	0.9800	C52—H52	0.9500
C16—H16A	0.9800	C53—H53	0.9500
C16—H16B	0.9800	C54—C56	1.515 (2)
C17—H17	0.9800	C54—C55	1.531 (2)
C17—H17A	0.9800	C54—H54	1.0000
C17—H17B	0.9800	C55—H55	0.9800
C18—C19	1.399 (2)	C55—H55A	0.9800
C18—C23	1.407 (2)	C55—H55B	0.9800
C19—C20	1.397 (2)	C56—H56	0.9800
C19—C24	1.505 (2)	C56—H56A	0.9800
C20—C21	1.380 (2)	C56—H56B	0.9800
C20—H20	0.9500	C57—H57	0.9800
C21—C22	1.387 (2)	C57—H57A	0.9800
C21—H21	0.9500	C57—H57B	0.9800
C22—C23	1.389 (2)	C58—C63	1.378 (3)
C22—H22	0.9500	C58—C59	1.382 (3)
C23—C25	1.504 (2)	C58—H58	0.9500
C24—H24	0.9800	C59—C60	1.364 (3)
C24—H24A	0.9800	C59—H59	0.9500
C24—H24B	0.9800	C60—C61	1.382 (3)
C25—H25	0.9800	C60—H60	0.9500
C25—H25A	0.9800	C61—C62	1.382 (3)
C25—H25B	0.9800	C61—H61	0.9500
C26—C27	1.383 (2)	C62—C63	1.372 (3)
C26—C31	1.3904 (19)	C62—H62	0.9500
C27—C28	1.393 (2)	C63—H63	0.9500
C27—H27	0.9500	C64—C65	1.372 (4)
C28—C29	1.403 (2)	C64—C69	1.380 (4)
C28—C32	1.496 (2)	C64—H64	0.9500
C29—C30	1.387 (2)	C65—C66	1.377 (3)
C29—H29	0.9500	C65—H65	0.9500
C30—C31	1.390 (2)	C66—C67	1.377 (3)
C30—C40	1.5035 (19)	C66—H66	0.9500
C31—H31	0.9500	C67—C68	1.382 (3)
C32—C37	1.397 (3)	C67—H67	0.9500
C32—C33	1.408 (2)	C68—C69	1.366 (3)
C33—C34	1.395 (3)	C68—H68	0.9500
C33—C38	1.506 (3)	C69—H69	0.9500
C34—C35	1.359 (3)		
			
C1—Ru1—C5	77.14 (6)	C32—C33—C38	122.87 (16)
C1—Ru1—C49	123.85 (6)	C35—C34—C33	121.3 (2)
C5—Ru1—C49	93.05 (6)	C35—C34—H34	119.4
C1—Ru1—C48	96.49 (6)	C33—C34—H34	119.4
C5—Ru1—C48	114.45 (5)	C34—C35—C36	120.16 (19)
C49—Ru1—C48	38.10 (5)	C34—C35—H35	119.9
C1—Ru1—C53	96.32 (6)	C36—C35—H35	119.9
C5—Ru1—C53	151.26 (5)	C35—C36—C37	120.8 (2)
C49—Ru1—C53	67.11 (5)	C35—C36—H36	119.6
C48—Ru1—C53	37.69 (5)	C37—C36—H36	119.6
C1—Ru1—C52	119.00 (6)	C32—C37—C36	118.96 (18)
C5—Ru1—C52	163.83 (6)	C32—C37—C39	122.19 (16)
C49—Ru1—C52	78.81 (6)	C36—C37—C39	118.81 (18)
C48—Ru1—C52	67.06 (6)	C33—C38—H38	109.5
C53—Ru1—C52	35.83 (6)	C33—C38—H38A	109.5
C1—Ru1—C50	161.33 (6)	H38—C38—H38A	109.5
C5—Ru1—C50	100.35 (6)	C33—C38—H38B	109.5
C49—Ru1—C50	37.49 (5)	H38—C38—H38B	109.5
C48—Ru1—C50	67.41 (5)	H38A—C38—H38B	109.5
C53—Ru1—C50	76.89 (5)	C37—C39—H39	109.5
C52—Ru1—C50	64.75 (6)	C37—C39—H39A	109.5
C1—Ru1—C51	154.86 (6)	H39—C39—H39A	109.5
C5—Ru1—C51	127.25 (6)	C37—C39—H39B	109.5
C49—Ru1—C51	66.65 (6)	H39—C39—H39B	109.5
C48—Ru1—C51	79.52 (6)	H39A—C39—H39B	109.5
C53—Ru1—C51	65.34 (6)	C41—C40—C45	121.78 (16)
C52—Ru1—C51	36.62 (6)	C41—C40—C30	119.48 (16)
C50—Ru1—C51	35.47 (6)	C45—C40—C30	118.56 (17)
C1—Ru1—Cl1	83.88 (4)	C40—C41—C42	118.3 (2)
C5—Ru1—Cl1	88.20 (4)	C40—C41—C46	121.48 (15)
C49—Ru1—Cl1	151.81 (4)	C42—C41—C46	120.2 (2)
C48—Ru1—Cl1	156.94 (4)	C43—C42—C41	119.8 (2)
C53—Ru1—Cl1	119.26 (4)	C43—C42—H42	120.1
C52—Ru1—Cl1	92.50 (4)	C41—C42—H42	120.1
C50—Ru1—Cl1	114.67 (4)	C44—C43—C42	121.42 (18)
C51—Ru1—Cl1	90.36 (4)	C44—C43—H43	119.3
N1—C1—N2	103.80 (13)	C42—C43—H43	119.3
N1—C1—Ru1	118.37 (10)	C43—C44—C45	120.5 (2)
N2—C1—Ru1	137.78 (11)	C43—C44—H44	119.7
C1—N1—C2	112.28 (12)	C45—C44—H44	119.7
C1—N1—C4	116.48 (12)	C44—C45—C40	118.1 (2)
C2—N1—C4	131.23 (13)	C44—C45—C47	119.8 (2)
C3—C2—N1	105.84 (14)	C40—C45—C47	122.06 (16)
C3—C2—H2	127.1	C41—C46—H46	109.5
N1—C2—H2	127.1	C41—C46—H46A	109.5
C2—C3—N2	107.57 (13)	H46—C46—H46A	109.5
C2—C3—H3	126.2	C41—C46—H46B	109.5
N2—C3—H3	126.2	H46—C46—H46B	109.5
C1—N2—C3	110.50 (12)	H46A—C46—H46B	109.5
C1—N2—C26	126.56 (13)	C45—C47—H47	109.5
C3—N2—C26	122.72 (12)	C45—C47—H47A	109.5
C9—C4—C5	126.27 (13)	H47—C47—H47A	109.5
C9—C4—N1	120.21 (13)	C45—C47—H47B	109.5
C5—C4—N1	113.52 (13)	H47—C47—H47B	109.5
C4—C5—C6	113.47 (13)	H47A—C47—H47B	109.5
C4—C5—Ru1	114.45 (10)	C49—C48—C53	117.32 (13)
C6—C5—Ru1	132.08 (11)	C49—C48—C54	123.09 (14)
C7—C6—C5	121.27 (13)	C53—C48—C54	119.55 (14)
C7—C6—C10	115.39 (12)	C49—C48—Ru1	69.88 (8)
C5—C6—C10	123.24 (13)	C53—C48—Ru1	73.37 (8)
C8—C7—C6	122.45 (13)	C54—C48—Ru1	129.30 (11)
C8—C7—H7	118.8	C48—C49—C50	120.92 (14)
C6—C7—H7	118.8	C48—C49—Ru1	72.02 (8)
C7—C8—C9	117.75 (13)	C50—C49—Ru1	75.75 (9)
C7—C8—C18	122.76 (13)	C48—C49—H49	119.5
C9—C8—C18	119.48 (13)	C50—C49—H49	119.5
C4—C9—C8	118.78 (14)	Ru1—C49—H49	124.2
C4—C9—H9	120.6	C51—C50—C49	120.49 (14)
C8—C9—H9	120.6	C51—C50—Ru1	73.07 (9)
C11—C10—C15	120.35 (15)	C49—C50—Ru1	66.76 (8)
C11—C10—C6	119.19 (14)	C51—C50—H50	119.8
C15—C10—C6	120.44 (14)	C49—C50—H50	119.8
C12—C11—C10	119.19 (16)	Ru1—C50—H50	133.7
C12—C11—C16	118.88 (15)	C50—C51—C52	118.84 (14)
C10—C11—C16	121.92 (14)	C50—C51—C57	121.45 (15)
C13—C12—C11	120.66 (16)	C52—C51—C57	119.67 (15)
C13—C12—H12	119.7	C50—C51—Ru1	71.46 (9)
C11—C12—H12	119.7	C52—C51—Ru1	70.75 (9)
C12—C13—C14	119.77 (16)	C57—C51—Ru1	127.87 (12)
C12—C13—H13	120.1	C53—C52—C51	120.40 (15)
C14—C13—H13	120.1	C53—C52—Ru1	71.00 (9)
C13—C14—C15	121.39 (16)	C51—C52—Ru1	72.63 (9)
C13—C14—H14	119.3	C53—C52—H52	119.8
C15—C14—H14	119.3	C51—C52—H52	119.8
C14—C15—C10	118.44 (15)	Ru1—C52—H52	128.9
C14—C15—C17	120.66 (15)	C52—C53—C48	121.62 (14)
C10—C15—C17	120.89 (15)	C52—C53—Ru1	73.17 (9)
C11—C16—H16	109.5	C48—C53—Ru1	68.94 (8)
C11—C16—H16A	109.5	C52—C53—H53	119.2
H16—C16—H16A	109.5	C48—C53—H53	119.2
C11—C16—H16B	109.5	Ru1—C53—H53	131.7
H16—C16—H16B	109.5	C48—C54—C56	113.15 (13)
H16A—C16—H16B	109.5	C48—C54—C55	109.44 (14)
C15—C17—H17	109.5	C56—C54—C55	110.97 (15)
C15—C17—H17A	109.5	C48—C54—H54	107.7
H17—C17—H17A	109.5	C56—C54—H54	107.7
C15—C17—H17B	109.5	C55—C54—H54	107.7
H17—C17—H17B	109.5	C54—C55—H55	109.5
H17A—C17—H17B	109.5	C54—C55—H55A	109.5
C19—C18—C23	120.48 (14)	H55—C55—H55A	109.5
C19—C18—C8	119.89 (13)	C54—C55—H55B	109.5
C23—C18—C8	119.62 (13)	H55—C55—H55B	109.5
C20—C19—C18	118.87 (14)	H55A—C55—H55B	109.5
C20—C19—C24	119.88 (14)	C54—C56—H56	109.5
C18—C19—C24	121.18 (14)	C54—C56—H56A	109.5
C21—C20—C19	120.92 (15)	H56—C56—H56A	109.5
C21—C20—H20	119.5	C54—C56—H56B	109.5
C19—C20—H20	119.5	H56—C56—H56B	109.5
C20—C21—C22	119.85 (15)	H56A—C56—H56B	109.5
C20—C21—H21	120.1	C51—C57—H57	109.5
C22—C21—H21	120.1	C51—C57—H57A	109.5
C21—C22—C23	120.95 (15)	H57—C57—H57A	109.5
C21—C22—H22	119.5	C51—C57—H57B	109.5
C23—C22—H22	119.5	H57—C57—H57B	109.5
C22—C23—C18	118.88 (14)	H57A—C57—H57B	109.5
C22—C23—C25	120.82 (14)	C63—C58—C59	119.8 (2)
C18—C23—C25	120.27 (14)	C63—C58—H58	120.1
C19—C24—H24	109.5	C59—C58—H58	120.1
C19—C24—H24A	109.5	C60—C59—C58	120.04 (19)
H24—C24—H24A	109.5	C60—C59—H59	120.0
C19—C24—H24B	109.5	C58—C59—H59	120.0
H24—C24—H24B	109.5	C59—C60—C61	120.4 (2)
H24A—C24—H24B	109.5	C59—C60—H60	119.8
C23—C25—H25	109.5	C61—C60—H60	119.8
C23—C25—H25A	109.5	C60—C61—C62	119.7 (2)
H25—C25—H25A	109.5	C60—C61—H61	120.1
C23—C25—H25B	109.5	C62—C61—H61	120.1
H25—C25—H25B	109.5	C63—C62—C61	119.85 (19)
H25A—C25—H25B	109.5	C63—C62—H62	120.1
C27—C26—C31	121.54 (14)	C61—C62—H62	120.1
C27—C26—N2	119.76 (12)	C62—C63—C58	120.25 (19)
C31—C26—N2	118.64 (13)	C62—C63—H63	119.9
C26—C27—C28	119.77 (13)	C58—C63—H63	119.9
C26—C27—H27	120.1	C65—C64—C69	120.1 (2)
C28—C27—H27	120.1	C65—C64—H64	119.9
C27—C28—C29	118.69 (14)	C69—C64—H64	119.9
C27—C28—C32	119.41 (13)	C64—C65—C66	120.8 (2)
C29—C28—C32	121.89 (14)	C64—C65—H65	119.6
C30—C29—C28	121.15 (14)	C66—C65—H65	119.6
C30—C29—H29	119.4	C67—C66—C65	118.9 (3)
C28—C29—H29	119.4	C67—C66—H66	120.6
C29—C30—C31	119.70 (13)	C65—C66—H66	120.6
C29—C30—C40	122.21 (14)	C66—C67—C68	120.3 (2)
C31—C30—C40	118.09 (14)	C66—C67—H67	119.9
C30—C31—C26	119.11 (14)	C68—C67—H67	119.9
C30—C31—H31	120.4	C69—C68—C67	120.5 (2)
C26—C31—H31	120.4	C69—C68—H68	119.8
C37—C32—C33	119.85 (16)	C67—C68—H68	119.8
C37—C32—C28	120.38 (15)	C68—C69—C64	119.4 (3)
C33—C32—C28	119.75 (16)	C68—C69—H69	120.3
C34—C33—C32	119.0 (2)	C64—C69—H69	120.3
C34—C33—C38	118.17 (18)		
			
C5—Ru1—C1—N1	1.61 (10)	C41—C40—C45—C44	−0.2 (3)
C49—Ru1—C1—N1	86.60 (12)	C30—C40—C45—C44	174.94 (16)
C48—Ru1—C1—N1	115.27 (11)	C41—C40—C45—C47	−178.45 (18)
C53—Ru1—C1—N1	153.21 (11)	C30—C40—C45—C47	−3.3 (3)
C52—Ru1—C1—N1	−177.40 (10)	C1—Ru1—C48—C49	−139.77 (9)
C50—Ru1—C1—N1	85.89 (19)	C5—Ru1—C48—C49	−60.98 (10)
C51—Ru1—C1—N1	−165.58 (11)	C53—Ru1—C48—C49	128.29 (14)
Cl1—Ru1—C1—N1	−87.93 (10)	C52—Ru1—C48—C49	101.43 (10)
C5—Ru1—C1—N2	178.56 (16)	C50—Ru1—C48—C49	30.44 (9)
C49—Ru1—C1—N2	−96.45 (16)	C51—Ru1—C48—C49	65.34 (9)
C48—Ru1—C1—N2	−67.78 (16)	Cl1—Ru1—C48—C49	130.62 (10)
C53—Ru1—C1—N2	−29.84 (16)	C1—Ru1—C48—C53	91.94 (10)
C52—Ru1—C1—N2	−0.45 (17)	C5—Ru1—C48—C53	170.74 (9)
C50—Ru1—C1—N2	−97.2 (2)	C49—Ru1—C48—C53	−128.29 (14)
C51—Ru1—C1—N2	11.4 (2)	C52—Ru1—C48—C53	−26.86 (9)
Cl1—Ru1—C1—N2	89.02 (15)	C50—Ru1—C48—C53	−97.85 (10)
N2—C1—N1—C2	0.20 (16)	C51—Ru1—C48—C53	−62.95 (9)
Ru1—C1—N1—C2	178.08 (10)	Cl1—Ru1—C48—C53	2.33 (17)
N2—C1—N1—C4	−179.11 (12)	C1—Ru1—C48—C54	−22.94 (15)
Ru1—C1—N1—C4	−1.22 (16)	C5—Ru1—C48—C54	55.86 (15)
C1—N1—C2—C3	0.40 (17)	C49—Ru1—C48—C54	116.84 (18)
C4—N1—C2—C3	179.58 (15)	C53—Ru1—C48—C54	−114.87 (18)
N1—C2—C3—N2	−0.82 (17)	C52—Ru1—C48—C54	−141.73 (16)
N1—C1—N2—C3	−0.71 (15)	C50—Ru1—C48—C54	147.27 (16)
Ru1—C1—N2—C3	−177.95 (12)	C51—Ru1—C48—C54	−177.83 (15)
N1—C1—N2—C26	173.97 (13)	Cl1—Ru1—C48—C54	−112.54 (15)
Ru1—C1—N2—C26	−3.3 (2)	C53—C48—C49—C50	−2.4 (2)
C2—C3—N2—C1	0.99 (18)	C54—C48—C49—C50	175.25 (14)
C2—C3—N2—C26	−173.93 (13)	Ru1—C48—C49—C50	−60.26 (13)
C1—N1—C4—C9	−179.43 (12)	C53—C48—C49—Ru1	57.84 (12)
C2—N1—C4—C9	1.4 (2)	C54—C48—C49—Ru1	−124.50 (15)
C1—N1—C4—C5	−0.36 (18)	C1—Ru1—C49—C48	50.59 (11)
C2—N1—C4—C5	−179.51 (14)	C5—Ru1—C49—C48	127.14 (9)
C9—C4—C5—C6	0.5 (2)	C53—Ru1—C49—C48	−31.39 (9)
N1—C4—C5—C6	−178.55 (12)	C52—Ru1—C49—C48	−66.95 (9)
C9—C4—C5—Ru1	−179.34 (11)	C50—Ru1—C49—C48	−129.78 (13)
N1—C4—C5—Ru1	1.66 (15)	C51—Ru1—C49—C48	−103.27 (10)
C1—Ru1—C5—C4	−1.75 (10)	Cl1—Ru1—C49—C48	−140.98 (8)
C49—Ru1—C5—C4	−125.81 (11)	C1—Ru1—C49—C50	−179.63 (8)
C48—Ru1—C5—C4	−93.10 (11)	C5—Ru1—C49—C50	−103.08 (9)
C53—Ru1—C5—C4	−81.29 (16)	C48—Ru1—C49—C50	129.78 (13)
C52—Ru1—C5—C4	175.14 (17)	C53—Ru1—C49—C50	98.39 (10)
C50—Ru1—C5—C4	−162.86 (10)	C52—Ru1—C49—C50	62.83 (9)
C51—Ru1—C5—C4	171.46 (9)	C51—Ru1—C49—C50	26.51 (8)
Cl1—Ru1—C5—C4	82.38 (10)	Cl1—Ru1—C49—C50	−11.20 (14)
C1—Ru1—C5—C6	178.51 (14)	C48—C49—C50—C51	6.8 (2)
C49—Ru1—C5—C6	54.46 (14)	Ru1—C49—C50—C51	−51.64 (13)
C48—Ru1—C5—C6	87.16 (14)	C48—C49—C50—Ru1	58.44 (13)
C53—Ru1—C5—C6	98.98 (16)	C1—Ru1—C50—C51	136.03 (17)
C52—Ru1—C5—C6	−4.6 (3)	C5—Ru1—C50—C51	−143.53 (9)
C50—Ru1—C5—C6	17.40 (14)	C49—Ru1—C50—C51	135.07 (13)
C51—Ru1—C5—C6	−8.28 (16)	C48—Ru1—C50—C51	104.16 (10)
Cl1—Ru1—C5—C6	−97.36 (13)	C53—Ru1—C50—C51	65.71 (9)
C4—C5—C6—C7	−0.39 (19)	C52—Ru1—C50—C51	29.85 (9)
Ru1—C5—C6—C7	179.34 (11)	Cl1—Ru1—C50—C51	−50.73 (9)
C4—C5—C6—C10	175.82 (13)	C1—Ru1—C50—C49	1.0 (2)
Ru1—C5—C6—C10	−4.4 (2)	C5—Ru1—C50—C49	81.41 (9)
C5—C6—C7—C8	0.3 (2)	C48—Ru1—C50—C49	−30.90 (9)
C10—C6—C7—C8	−176.21 (13)	C53—Ru1—C50—C49	−69.36 (9)
C6—C7—C8—C9	−0.2 (2)	C52—Ru1—C50—C49	−105.22 (10)
C6—C7—C8—C18	178.86 (13)	C51—Ru1—C50—C49	−135.07 (13)
C5—C4—C9—C8	−0.4 (2)	Cl1—Ru1—C50—C49	174.20 (7)
N1—C4—C9—C8	178.58 (13)	C49—C50—C51—C52	−5.6 (2)
C7—C8—C9—C4	0.2 (2)	Ru1—C50—C51—C52	−54.43 (13)
C18—C8—C9—C4	−178.87 (13)	C49—C50—C51—C57	172.49 (15)
C7—C6—C10—C11	77.39 (17)	Ru1—C50—C51—C57	123.63 (16)
C5—C6—C10—C11	−99.03 (18)	C49—C50—C51—Ru1	48.87 (13)
C7—C6—C10—C15	−101.14 (17)	C1—Ru1—C51—C50	−148.47 (12)
C5—C6—C10—C15	82.45 (18)	C5—Ru1—C51—C50	47.28 (11)
C15—C10—C11—C12	−4.5 (2)	C49—Ru1—C51—C50	−27.91 (9)
C6—C10—C11—C12	176.97 (13)	C48—Ru1—C51—C50	−65.56 (9)
C15—C10—C11—C16	174.15 (14)	C53—Ru1—C51—C50	−102.37 (10)
C6—C10—C11—C16	−4.4 (2)	C52—Ru1—C51—C50	−131.00 (14)
C10—C11—C12—C13	1.0 (2)	Cl1—Ru1—C51—C50	135.29 (8)
C16—C11—C12—C13	−177.74 (15)	C1—Ru1—C51—C52	−17.47 (18)
C11—C12—C13—C14	2.8 (2)	C5—Ru1—C51—C52	178.28 (8)
C12—C13—C14—C15	−3.0 (2)	C49—Ru1—C51—C52	103.09 (10)
C13—C14—C15—C10	−0.5 (2)	C48—Ru1—C51—C52	65.44 (10)
C13—C14—C15—C17	178.53 (16)	C53—Ru1—C51—C52	28.63 (9)
C11—C10—C15—C14	4.3 (2)	C50—Ru1—C51—C52	131.00 (14)
C6—C10—C15—C14	−177.23 (14)	Cl1—Ru1—C51—C52	−93.71 (9)
C11—C10—C15—C17	−174.75 (15)	C1—Ru1—C51—C57	95.66 (19)
C6—C10—C15—C17	3.8 (2)	C5—Ru1—C51—C57	−68.59 (17)
C7—C8—C18—C19	−86.40 (18)	C49—Ru1—C51—C57	−143.79 (16)
C9—C8—C18—C19	92.61 (17)	C48—Ru1—C51—C57	178.57 (16)
C7—C8—C18—C23	94.86 (18)	C53—Ru1—C51—C57	141.76 (17)
C9—C8—C18—C23	−86.13 (17)	C52—Ru1—C51—C57	113.13 (19)
C23—C18—C19—C20	2.4 (2)	C50—Ru1—C51—C57	−115.87 (19)
C8—C18—C19—C20	−176.33 (13)	Cl1—Ru1—C51—C57	19.42 (15)
C23—C18—C19—C24	−174.46 (13)	C50—C51—C52—C53	0.2 (2)
C8—C18—C19—C24	6.8 (2)	C57—C51—C52—C53	−177.94 (15)
C18—C19—C20—C21	−1.3 (2)	Ru1—C51—C52—C53	−54.62 (14)
C24—C19—C20—C21	175.61 (14)	C50—C51—C52—Ru1	54.77 (13)
C19—C20—C21—C22	−0.3 (2)	C57—C51—C52—Ru1	−123.32 (15)
C20—C21—C22—C23	0.7 (2)	C1—Ru1—C52—C53	−56.44 (11)
C21—C22—C23—C18	0.4 (2)	C5—Ru1—C52—C53	127.03 (18)
C21—C22—C23—C25	−177.69 (14)	C49—Ru1—C52—C53	66.21 (10)
C19—C18—C23—C22	−2.0 (2)	C48—Ru1—C52—C53	28.15 (9)
C8—C18—C23—C22	176.77 (13)	C50—Ru1—C52—C53	102.99 (10)
C19—C18—C23—C25	176.13 (14)	C51—Ru1—C52—C53	131.95 (14)
C8—C18—C23—C25	−5.1 (2)	Cl1—Ru1—C52—C53	−140.82 (9)
C1—N2—C26—C27	−46.2 (2)	C1—Ru1—C52—C51	171.62 (9)
C3—N2—C26—C27	127.85 (16)	C5—Ru1—C52—C51	−4.9 (2)
C1—N2—C26—C31	136.38 (15)	C49—Ru1—C52—C51	−65.73 (9)
C3—N2—C26—C31	−49.5 (2)	C48—Ru1—C52—C51	−103.79 (10)
C31—C26—C27—C28	−0.6 (2)	C53—Ru1—C52—C51	−131.94 (14)
N2—C26—C27—C28	−177.95 (14)	C50—Ru1—C52—C51	−28.96 (9)
C26—C27—C28—C29	1.9 (2)	Cl1—Ru1—C52—C51	87.23 (9)
C26—C27—C28—C32	−178.81 (14)	C51—C52—C53—C48	4.2 (2)
C27—C28—C29—C30	−1.8 (2)	Ru1—C52—C53—C48	−51.15 (13)
C32—C28—C29—C30	178.88 (16)	C51—C52—C53—Ru1	55.38 (14)
C28—C29—C30—C31	0.5 (3)	C49—C48—C53—C52	−3.0 (2)
C28—C29—C30—C40	−178.92 (16)	C54—C48—C53—C52	179.21 (15)
C29—C30—C31—C26	0.8 (2)	Ru1—C48—C53—C52	53.02 (14)
C40—C30—C31—C26	−179.79 (15)	C49—C48—C53—Ru1	−56.05 (12)
C27—C26—C31—C30	−0.7 (2)	C54—C48—C53—Ru1	126.19 (14)
N2—C26—C31—C30	176.63 (14)	C1—Ru1—C53—C52	132.84 (10)
C27—C28—C32—C37	122.23 (17)	C5—Ru1—C53—C52	−152.46 (12)
C29—C28—C32—C37	−58.5 (2)	C49—Ru1—C53—C52	−102.99 (10)
C27—C28—C32—C33	−56.3 (2)	C48—Ru1—C53—C52	−134.71 (14)
C29—C28—C32—C33	122.98 (18)	C50—Ru1—C53—C52	−64.81 (10)
C37—C32—C33—C34	1.4 (2)	C51—Ru1—C53—C52	−29.22 (9)
C28—C32—C33—C34	179.90 (16)	Cl1—Ru1—C53—C52	46.34 (10)
C37—C32—C33—C38	−178.61 (18)	C1—Ru1—C53—C48	−92.45 (10)
C28—C32—C33—C38	−0.1 (3)	C5—Ru1—C53—C48	−17.75 (17)
C32—C33—C34—C35	−0.1 (3)	C49—Ru1—C53—C48	31.71 (9)
C38—C33—C34—C35	179.9 (2)	C52—Ru1—C53—C48	134.71 (14)
C33—C34—C35—C36	−1.1 (3)	C50—Ru1—C53—C48	69.90 (9)
C34—C35—C36—C37	0.9 (3)	C51—Ru1—C53—C48	105.49 (10)
C33—C32—C37—C36	−1.5 (2)	Cl1—Ru1—C53—C48	−178.95 (8)
C28—C32—C37—C36	179.97 (14)	C49—C48—C54—C56	31.6 (2)
C33—C32—C37—C39	176.19 (16)	C53—C48—C54—C56	−150.78 (15)
C28—C32—C37—C39	−2.3 (2)	Ru1—C48—C54—C56	−58.6 (2)
C35—C36—C37—C32	0.4 (3)	C49—C48—C54—C55	−92.70 (18)
C35—C36—C37—C39	−177.39 (18)	C53—C48—C54—C55	84.92 (19)
C29—C30—C40—C41	−99.8 (2)	Ru1—C48—C54—C55	177.14 (12)
C31—C30—C40—C41	80.8 (2)	C63—C58—C59—C60	−0.2 (3)
C29—C30—C40—C45	85.0 (2)	C58—C59—C60—C61	0.2 (3)
C31—C30—C40—C45	−94.5 (2)	C59—C60—C61—C62	−0.4 (3)
C45—C40—C41—C42	−1.6 (3)	C60—C61—C62—C63	0.5 (3)
C30—C40—C41—C42	−176.67 (16)	C61—C62—C63—C58	−0.5 (3)
C45—C40—C41—C46	177.65 (18)	C59—C58—C63—C62	0.3 (3)
C30—C40—C41—C46	2.5 (3)	C69—C64—C65—C66	−0.5 (4)
C40—C41—C42—C43	2.0 (3)	C64—C65—C66—C67	0.4 (4)
C46—C41—C42—C43	−177.2 (2)	C65—C66—C67—C68	0.2 (3)
C41—C42—C43—C44	−0.6 (3)	C66—C67—C68—C69	−0.5 (3)
C42—C43—C44—C45	−1.2 (3)	C67—C68—C69—C64	0.3 (4)
C43—C44—C45—C40	1.6 (3)	C65—C64—C69—C68	0.2 (4)
C43—C44—C45—C47	179.9 (2)		
