# Supplementary figures and images for: Crystal structure of {3-[3,5-bis­(2,6-di­methyl­phen­yl)-1,2-phenyl­ene]-1-(2,6,2′′,6′′-tetra­methyl-1,1′:3′,1′′-ter­phen­yl-5′-yl)imidazol-2-yl­idene}chlorido­(η6-p-cymene)ruthenium(II) benzene disolvate
Source: Acta Crystallogr Sect E Struct Rep Online. 2014 Nov 8;70(Pt 12):m394. doi: 10.1107/S160053681402399X (PMC4257382; doi:10.1107/S160053681402399X)

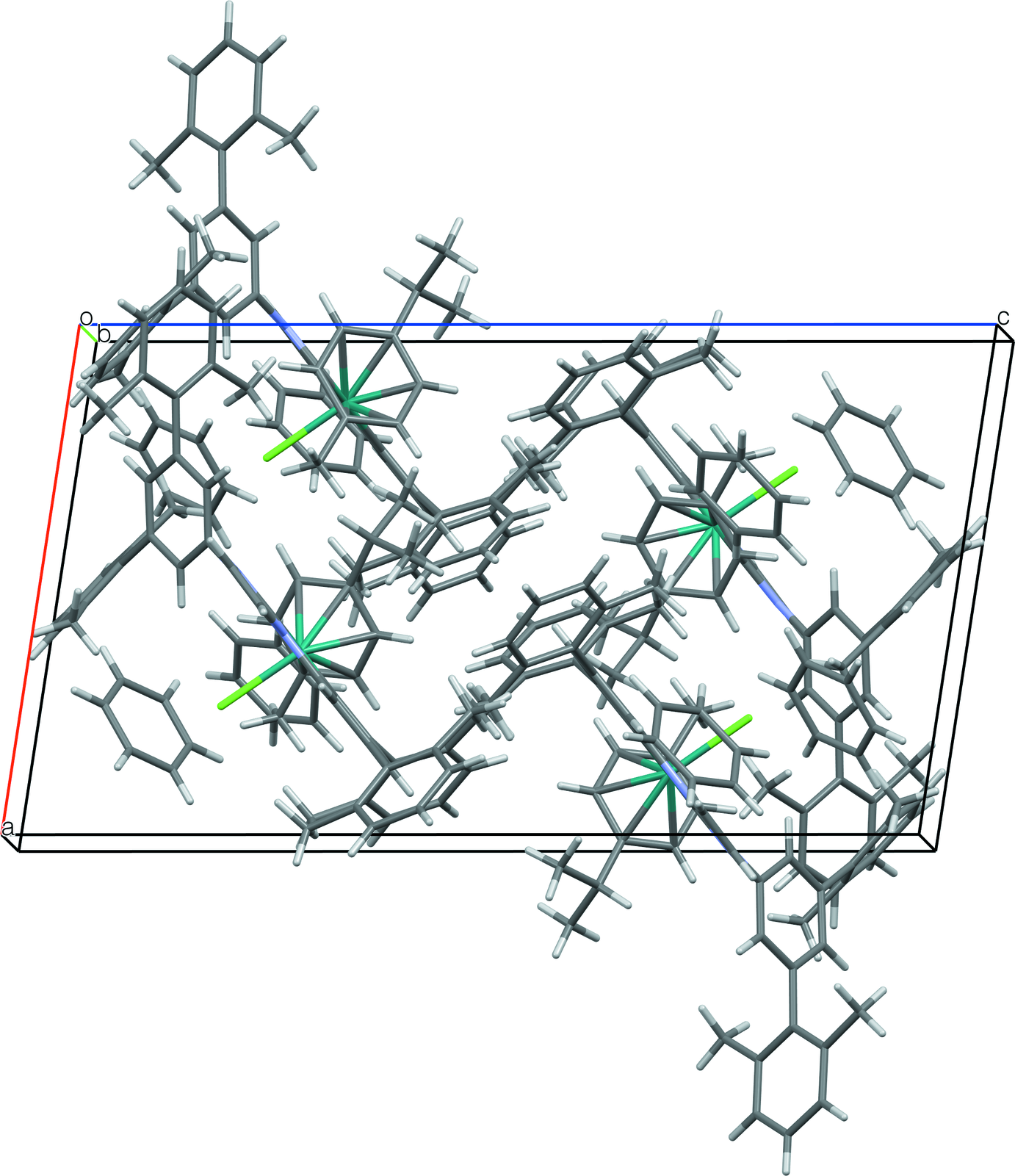

Supplement: Supplementary file 4 [file e-70-0m394-fig2.tif]
